# Pregnancy after orthotopic liver transplantation: a comprehensive review

**DOI:** 10.3389/frtra.2025.1581273

**Published:** 2025-06-06

**Authors:** Daria A. Stelmach, Kenneth J. Dery, Zoulikha Jabiry-Zieniewicz, Jerzy W. Kupiec-Weglinski

**Affiliations:** ^1^ Dumont-UCLA Transplantation Center, Division of Liver and Pancreas Transplantation, Department of Surgery, David Geffen School of Medicine at UCLA, Los Angeles, CA, United States; ^2^ First Department of Obstetrics and Gynecology, Medical University of Warsaw, Warsaw, Poland

**Keywords:** pregnancy after liver transplantation, family planning in liver transplant recipients, post-transplant pregnancy risks, immunosuppression during pregnancy, liver transplant and maternal outcomes, liver transplant and neonatal outcomes

## Abstract

**Background:**

Medical innovations and advancements, such as orthotopic liver transplantation (OLT) allow thousands of patients worldwide to live comfortably, despite previously life-threatening conditions. Procreation, one of the most powerful human instincts, drives the force behind the increasing popularity of pregnancies after OLT, with their numbers rising since the first documented case in 1976. Pregnancy post OLT remains a high-risk event, requiring careful management by a multidisciplinary team of hepatologists, obstetricians, transplant surgeons, and neonatologists. This review aims to synthesize current evidence on family planning, pregnancy management, and maternal and neonatal outcomes in women who have undergone OLT, based on studies indexed in PubMed up to December 2024.

**Findings:**

Due to ethical constraints, international registries of pregnancies after OLTs play a critical role in collecting observational data and establishing comprehensive guidelines for clinical practice. As the data indicated, OLT can help restore hormonal balance and menstrual cycle, enabling many women to conceive after OLT. However, adequate family planning is crucial, as women must be aware of the potential risks. Preconception counseling is essential to choose the right timing for pregnancy, assess graft function, and optimize immunosuppressive therapy, as some medications must be discontinued due to teratogenic risks. The risks associated with pregnancy in OLT recipients include gestational hypertension, preeclampsia, and gestational diabetes. Neonates are significantly more likely to experience prematurity and low birth weight. Post-partum management focuses on monitoring graft function, managing complications, and guiding breastfeeding.

**Conclusions:**

Available literature and observational studies consistently demonstrate that women post-OLT can achieve successful pregnancies and deliver healthy infants. However, due to the inherent risks described in this population, such patients require specialized care from a multidisciplinary team. Further research is essential to optimize birth control methods and clarify the mechanisms behind the higher prevalence of pregnancy complications. Establishing the long-term safety data for immunosuppressive therapies, particularly regarding breastfeeding, is also needed.

## 1 Introduction

Advances in medical science have been providing new therapeutic opportunities, allowing physicians to treat previously incurable conditions. One of these great opportunities is organ transplantation, which significantly improves patient health and offers the possibility of survival and return to normal life. The first successful orthotopic liver transplantation (OLT) took place in 1967, and according to recent data, there were 37,436 OLTs performed in 2022 worldwide (1, 2). Trends show these numbers are increasing with no disparity between males and females among recipients across all age groups. According to Organ Procurement and Transplantation Network registries, between 2020 and 2024, only in the United States, there were more than 1,000 OLTs performed on average every year in females aged 18–49 (3).

OLT gives them a chance for fertility recovery, conception, and delivery of a healthy infant. The first successful pregnancy in OLT recipient was reported in 1976 (4). Even after 48 years of documented cases in the literature and numerous studies published on the subject, pregnancy after OLT is still considered a high-risk event that should be carefully monitored. It requires increased attention and care provided by the multidisciplinary medical team, typically including hepatologists, obstetricians, transplant surgeons, and neonatologists.

We conducted an extensive narrative review using the PubMed database to identify relevant studies on family planning and pregnancy following OLT. Review provides published data on fertility restoration, contraceptive measures, assisted reproductive technology, pre-pregnancy counseling, pregnancy, intrapartum and post-partum management, immunosuppression, maternal and neonatal complications, and breastfeeding options. Approximately 75% of the included studies, published in the years 2006–2024, reflect recent advancements, while the remaining 25% are landmark studies providing foundational knowledge in the field. Notably, about 50% of the studies were published in the last 10 years (2015–2024), highlighting the growing interest in this field. Only articles written in English were considered. The search terms included: “pregnancy' and “liver transplantation'; “pregnancy post-transplantation'; “pregnancy post-liver transplantation'; “contraception' and “transplant recipients'; “breastfeeding' and “transplantation'; “immunosuppression' and “pregnancy'; “immunosuppression' and “breastfeeding'; “*in vitro* fertilization' and “transplantation'. Due to the limited availability of data specific to OLT, this review includes studies on outcomes across all solid organ transplantations. Reliance on published studies and registry data, such as the Transplantation Pregnancy Registry International (TPRI), made it challenging to exclude overlapping cases, but the diversity of study designs, geographical regions, and time periods suggests minimal impact from potential double reporting.

## 2 Registries and current standards of post-OLT pregnancy care

Pregnancy after liver transplantation is becoming more common, yet not many centers in the world have considerable experience in the field. For obvious ethical and practical reasons, our knowledge, and recommendations on how to deal with pregnant women are solely based on observations. Therefore, special registers have been established to collect clinical data to determine the preferred course of action, resulting in the best outcomes for both the mother and the child. The leading catalog is Transplantation Pregnancy Registry International (TPRI), formerly known as National Transplantation Pregnancy Registry (NTPR) created in Philadelphia in 1991, which brings together information on more than 3,000 organ recipients (5). There was also a one-off effort from the United Kingdom: the UK Transplant Pregnancy Register, which covered the years 1994–2001 (6, 7). Moreover, there are several single and multi-center studies, series and single case reports published. More organized data concerning kidney recipients is available, including the European Dialysis and Transplant Association Registry and the Australian and New Zealand Dialysis and Transplant Registry (8, 9).

Extensive guidelines and recommendations were developed based on retrospectively collected records. The clinical approach to post-OLT patients is based on documents established by world-leading societies, such as American Association for the Study of Liver Diseases (AASLD) and their “Reproductive Health and Liver Disease: Practice Guidance” (10), Society for Maternal-Fetal Medicine with “Consult Series #66: Pre-pregnancy evaluation and pregnancy management of patients with solid organ transplants' by (11) and American Society of Transplantation (AST) with Consensus Conference on Reproductive Issues and Transplantation. These guidelines provide the most comprehensive and up-to-date recommendations (12).

## 3 OLT and fertility

Abnormal liver function is associated with sexual dysfunction, often caused by dysregulation of sex hormone metabolism, suppression of the hypothalamus-pituitary-ovary (HPO) axis, portal hypertension, medication, and underlying primary disease. Emotional and psychological factors also contribute significantly. The overall pathogenesis is complex and not fully understood (13). Impairment of liver function leads to increased aromatization of androgens to estrogen and elevated levels of sex hormone-binding globulins (SHBG), which results in lower availability of circulating free fraction of sex hormones. Therefore, the hormonal axes between the hypothalamus, pituitary and ovaries may become impaired, as they affect one another (14). This results in menstrual cycle abnormalities among females of reproductive age with liver failure, with amenorrhea being the most common disturbance, affecting 30%–70% of patients (15, 16). Interestingly, studies have shown that within one year post-OLT, 70%–95% of women reported a return to regular menstrual cycles. This resumption correlates with the stabilization of transplanted liver function and hormonal balance recovery.

A systematic review by Gariani et al., including 21 studies with a clinical cohort of 1,274 patients, revealed that because of OLT regaining its function, plasma levels of SHBG and sex hormones (testosterone and estradiol) were normal. Consequently, pituitary-secreted hormone levels (follicle-stimulating hormone and luteinizing hormone) were also found to normalize, supporting the idea that OLT impacts HPO axis recovery (17). In addition, 72% of females reported returning to sexual activity post-OLT (15, 16). Due to the fertility recovery and sexual activity by females in reproductive age, we can assume that adequate contraception and preconception counselling are essential for women in this group.

## 4 Contraceptive choices for OLT patients

The American Society of Transplantation (AST) recommends that women with pre-existing liver conditions receive specialized contraceptive counselling ahead of the transplant surgery procedure. Patients should be informed about the available methods, as there is no universally ideal option. The choice of contraception should be guided by individual factors, including risks, benefits, cost-effectiveness, future family planning intentions, and patient compliance. Many recipients opt for barrier methods, such as condoms, cervical caps, or diaphragms, as these methods do not interact with immunosuppressive therapy or compromise graft function. However, failure rates range from 13% for male condoms to 23% for cycle monitoring. Female sterilization or male vasectomy may be considered in cases where there is no desire for future pregnancies (18).

The American Center for Disease Control and Prevention (CDC) issued extensive recommendations regarding birth control—“U.S. Medical Eligibility Criteria for Contraceptive Use” (19). They divide different methods into four categories, indicating possible risks for patients with various conditions, which is detailed in Table 1.

**Table 1:** Contraception considerations for post-OLT patients. Adapted from CDC—U.S. Medical Eligibility Criteria for Contraceptive Use, 2024 (19).

Category/Method		Combined Hormonal Contraception	Progestin Only Pills	Copper-IUD (Cu-IUD)		Levonorgestrel-IUD		Progesterone Implant	DMPA	Barrier Methods
				Initiation	Continuation	Initiation	Continuation			
**CDC Category**	**Accurate graft functions**	2	2	1	1	1	1	1	2/3	1
	**Graft failure**	4	2	2	1	2	1	2	2/3	.1
**Considerations for post-OLT patients**		Not recommended with Budd-Chiari syndrome prior to OLT due to higher thrombosis risk. Estrogen-containing contraceptives may increase the risk of thrombosis and hypertension, especially during cyclosporine therapy.	Safer option due to the lack of estrogen effects. Possible interactions with immunosuppressive agents should be considered	Cu-IUDS might increase the risk of excessive bleeding. No hormonal effects; suitable for most patients. Low risk of infection, but should be monitored in immunocompromised patients		Minimal hormonal systemic effects. May help manage heavy menstrual bleeding. Low risk of infection, but should be monitored in immunocompromised patients.		Highly effective with minimal drug interactions. No significant impact on graft function.	Not advised for patients with risk factors or on long-term immunosuppression, particularly corticosteroids (Category 3). Bone health should be monitored in such patients	No hormonal effect or drug interactions. Reduces risk of sexually transmitted infections, important for immunocompromised patients.

Legend: Category 1, Method that is safe and there are no risks; Category 2, Method with the benefits that outweigh potential or proven risks; Category 3, Method that requires reconsideration because the risks may outweigh the advantages; Category 4, Methods that should not be applied due to unacceptable health hazards.

### 4.1 Combined hormonal contraception (CHC)

Combined hormonal contraception includes pills, transdermal patches and vaginal rings composed of estrogen and progestin. The mean failure rate for CHC is approximately 9%. However, CHCs increase the risk of deep vein thrombosis and stroke. Therefore, they should never be used in females with an ongoing or history of deep vein thrombosis or pulmonary embolism. Similarly, recipients with underlying Budd-Chiari syndrome must be discouraged from CHC use due to their elevated thromboembolism risk (19, 20). According to a study on 16 OLT recipients by Jabiry-Zieniewicz et al., no cases of pregnancy and rejection episodes were observed in a group of patients treated with low-dose combined hormonal contraception. Biochemical parameters of the hepatocellular function after OLT, fasting glucose levels and vital signs were assessed every three months during the first year of therapy. No significant abnormalities in biochemical parameters were obtained (21). Based on these findings, the AASLD considers CHC safe for OLT recipients, only if graft function is stable and hepatic parameters are within normal range (10).

### 4.2 Progestin-based therapies

Progestin-based therapies include progesterone-only pills, injected depot medroxyprogesterone acetate (DMPA), hormonal intrauterine device (IUD) and subcutaneous implants that slowly release levonorgestrel. The failure rate for the progesterone-only pill is approximately 9%, for DMPA 6%, and for implants 0.05% (22). Implants should be replaced every three years, and DMPA injections are given every 12 weeks. These options are considerably safe due to the absence of estrogen-driven effects (10). One study suggests that DMPA use may be associated with an increased likelihood of bone fractures (23). Therefore, CDC guidelines emphasize that its use should be reconsidered in OLT recipients who already are at risk of osteoporosis due to hormonal imbalance and immunosuppression (19). No studies evaluating liver function impairment due to progestin contraceptive therapy were found.

### 4.3 Intrauterine devices (IUD)

There are two types of intrauterine devices—copper and hormonal (levonorgestrel-based), both with failure risk lower than 1% (22). Although the American Society of Transplantation (AST) recommendations from 2005 were against IUD use in graft recipients, current Centers for Disease Control and Prevention guidelines are in favor of this method, claiming it is effective in terms of contraception and safe for the graft (19). There are no randomized trials regarding its use, and all the data are case-based only (24). According to a review by Paulen et al., three pregnancies occurred in analyzed literature regardless of the presence of IUD (25). Therefore, further studies and reviews have confirmed their effectiveness and safety across various graft conditions (26). Currently, they are also recommended as one of the first-line contraceptives by the American College of Obstetricians and Gynecologists for adolescents (27). Non-hormonal IUDs escalate menstrual bleeding, which should be taken into consideration before prescribing them to a patient post-OLT, as they are at risk of coagulopathies. Hormonal IUDs may have therapeutic applications for managing heavy menstrual bleeding, hemostatic disorders or pelvic pain (28). The insertion of the IUD, a foreign body placed in the uterus, raises concerns about its use in immunocompromised women due to a potential increased risk of infection (25). However, there is no significant literature supporting this correlation.

## 5 Preconception strategies for OLT recipients

**Figure 1:**
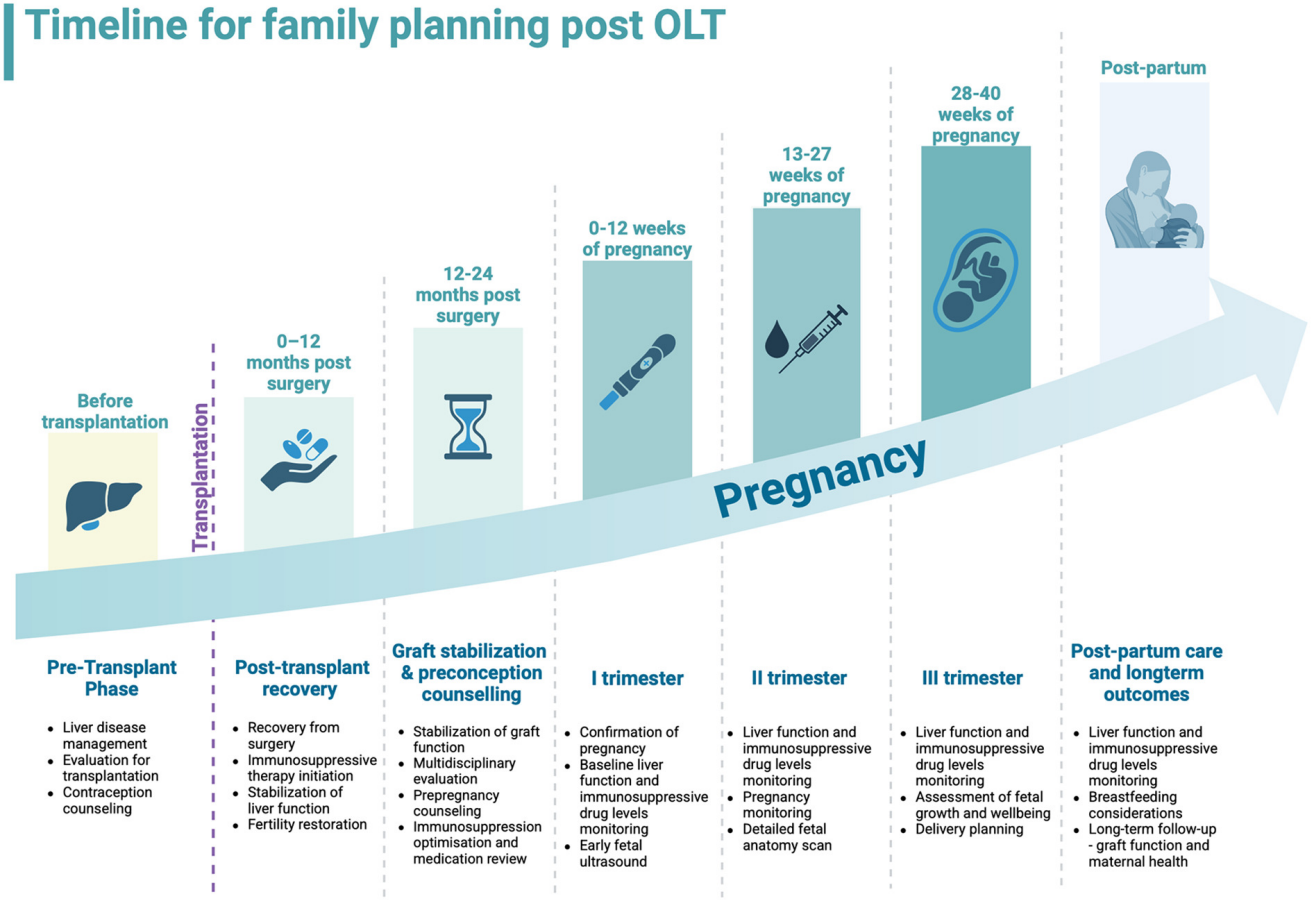
Timeline for family planning after orthotopic liver transplantation, from pre-transplant phase to post-partum. Created with BioRender.com.

Liver transplant patients regain the ability to conceive within weeks after surgery. Since immediate pregnancy is not advisable, thorough counselling is crucial. A survey by McIntosh et al. found that 38.8% of women who underwent solid organ transplantation (liver, kidney, and heart) considered conception, while 45.3% utilized some method of birth control. Among these women, 78.1% discussed conception plans with their physicians within three months post-transplantation and these findings are relevant to OLT due to similar post-transplant care protocols (29). The American Society of Transplantation (AST) created a joint statement regarding the reproductive health of patients after OLTs. Before attempting conception, certain requirements that must be met, including no acute rejection episodes within the past year, stable graft function, adequate immunosuppressive therapy, and absence of infection that could harm the fetus (12). Although as already mentioned, pregnancy in OLT recipients is classified as high-risk, 40% of pregnancies among this population in the United States remain unplanned and followed by increased risk of maternal and neonatal complications (30). The timeline for family planning is summarized in Figure 1 (31).

Pregnancy poses a challenge even for healthy individuals. Therefore, transplant recipients with a history of surgeries, impaired organ basal function and years of aggressive immunosuppressive therapy are categorized as high-risk groups of obstetric patients. Conception planning is crucial, as unplanned pregnancies among transplant recipients are associated with poorer maternal and neonatal outcomes (30). An experienced team of specialists should take the lead. A questionnaire-based study by MN Rahim et al. showed that patients who attended pre-pregnancy counseling found it beneficial, as it reassured them and helped them pursue the decision to have a baby (32). It is advised for medical providers to discuss possible fetal and maternal complications with prospective parents. Women should be made aware that pregnancy may affect liver function, and the risk for preterm delivery is elevated. Immunosuppressive regimens also require evaluation, as some medications may need dosage adjustments or discontinuation before conception.

The Society for Maternal-Fetal Medicine has developed a set of guidelines for the primary assessment of all transplant recipients who plan to conceive, with additional organ-specific recommendations detailing unique considerations. The assessment involves evaluation of the patient's medical records, including underlying disease, comorbidities, and post-transplant period. The current medication regimen should also be thoroughly evaluated. If therapy includes mycophenolic acid (MPA)-based agents, they should be replaced at least six weeks before conception due to teratogenic risks. Additionally, a physical examination, including blood pressure measurement and laboratory tests, is essential. Solid organ transplant recipients face an increased risk of red blood cell autoimmunization, which makes blood type assessment crucial. Psychological and social factors should also be considered (11).

Vaccination is also a vital component of preconception care in OLT recipients due to chronic immunosuppression. Women should receive inactivated vaccines, such as those for pneumococcus, hepatitis A and B, tetanus-diphtheria-pertussis, Haemophilus influenzae type B, human papillomavirus, seasonal influenza, and SARS-CoV-2, ideally completed prior to transplantation or during stable graft function periods as per AST guidelines (33). Live attenuated vaccines (measles-mumps-rubella, varicella-zoster virus) are contraindicated post-transplant due to the risk of vaccine-derived infection in immunocompromised patients (11, 33). Although in rare cases, live vaccines may be considered pre-transplant or under strict medical supervision with a risk-benefit analysis, this is not routine post-OLT practice.

Due to chronic immunosuppressive therapy, there is a high risk of developing opportunistic infections. Therefore, the patient's cytomegalovirus (CMV) immunological status should be tested before conception due to its frequent occurrence and possible harm to the fetus (11). Folic acid supplementation is strongly advised, with standard dosing guidelines applying—400 micrograms per day, unless the future mother does not present any indication for an increased dose. According to recommendations of the American College of Obstetricians and Gynecologists and the American Academy of Pediatrics, women's diet requires evaluation in terms of its content of protein and other nutrients such as calcium, iron, vitamin A, vitamin B12, vitamin B and vitamin D. Standard pregnancy supplements may be recommended for graft recipients (33, 34).

## 6 Assisted reproduction in OLT recipients

Fertility may be affected in OLT recipients due to persistent hormonal imbalances, chronic medication adverse effects, or the underlying conditions that provoked the transplantation. Moreover, past surgeries may result in intraabdominal adhesions, constituting a mechanical obstacle (35, 36). Although most recipients regain reproductive capabilities, there is a group of women who would fail to conceive.

The World Health Organization's definition of infertility assumes no conception after 12 months of regular intercourse and requires medical evaluation (37). In general, infertility management among graft recipients does not differ from the approach used in the general population; thus, recommended therapy depends on the diagnosis. Based on that, induction of ovulation, insemination or IVF might be suggested. Greater attention should be given to prevent complications, especially multiple pregnancies and ovarian hyperstimulation syndrome (OHSS), which may affect the transplanted organ. Another critical consideration is the potential inheritance of the underlying disease that led to organ transplantation, which should be discussed before initiating ART.

Data on *in vitro* fertilization (IVF) following OLT are very limited. However, there are known cases reporting favorable outcomes of IVFs and pregnancy without graft impairment. Described IVF procedures followed standard protocols, and graft functions were closely monitored throughout, including the time of ovarian stimulation (38, 39). One complex study included 11 patients who underwent 14 IVF cycles post OLT. All of them received tacrolimus as immunosuppressive therapy, two also taking prednisolone and one sirolimus. Liver enzymes increase was observed in three patients, and one person was diagnosed with OHSS and obstetric cholestasis. Among 14 analyzed IVF cycles, three failed in the implantation stage, two resulted in miscarriages, one stillbirth occurred, and five babies were delivered pre-term. Despite these complications, no cases of graft rejection or transplant loss were reported (36). Reproductive aspirations are a fundamental part of human health and should be addressed with appropriate care and respect. While Assisted Reproductive Technology is feasible in OLT recipients, it requires a carefully tailored approach and multidisciplinary care (40, 41).

## 7 Pregnancy care after OLT

Pregnancy induces significant physiological transformations in the female body to support fetal development and well-being. These adaptations affect all major systems, yet the cardiovascular system transforms most. Hormone changes affect endothelium function, which results in peripheral vasodilatation, reduced vascular resistance and increased cardiac output (42). As blood plasma volume increases, red blood cell count, hemoglobin concentration and hematocrit drop. Furthermore, glucose, lipids, proteins, and water metabolism shift, with the liver playing a pivotal role (43). Kidneys also adapt to the increased systemic blood volume and vasodilation, with results in renal plasma flow rising to 80%. Consequently, glomerular filtration rates increase, imposing greater fluid-handling demands on the kidneys. During gestation, they enlarge, and serum levels of creatinine, urea, and uric acid drop compared to non-pregnant females (44).

In terms of pregnancy management, routine visits should occur more frequently, at least every 4 weeks, to assess the fetus's well-being and the mother's vital signs, including blood pressure, heart rate and weight. Routine urine and laboratory tests should include blood counts, liver and kidney function markers (aspartate aminotransferase, alanine aminotransferase, bilirubin level, and creatinine), and immunosuppressive drug levels (tacrolimus/cyclosporine) for dose adjustments (11). A deep dive into the dose adjustment will be discussed later in a dedicated section of this review.

The initial pregnancy visit should encompass a detailed overview of medical history, physical exam, and vital signs measurement, followed by pap smear collection and serologic screening for CMV, Hepatitis B, Hepatitis C, HIV, syphilis and toxoplasmosis. Serology tests are performed within the first trimester and repeated, if negative, during the second and third ones. Blood type should be obtained, and those with rhesus-negative may require immunization prevention later in pregnancy. Vaccinations, including Tdap (administered between 27 and 36 weeks), seasonal influenza, and SARS-CoV-2, are strongly recommended. Prior transplantation is not a contraindication (11).

As transplant recipients are at high risk for diabetes mellitus, a routine screening test is advised earlier than 24–28 weeks, depending on locally applied recommendations (35, 45). Ultrasound assessments follow general pregnancy guidelines, with nuchal translucency (NT) and anatomy scans recommended at 11 + 0−13 + 6 weeks and 18 + 0−21 + 6 weeks, respectively (46). Rahim et al. suggest performing additional ultrasounds to assess fetal growth at 28, 32 and 36 weeks followed by middle cerebral and umbilical artery Doppler, if fetal growth restriction (FGR) is suspected (35). Non-invasive prenatal testing (NIPT), assessing cell-free fetal DNA (cffDNA), is a recommended screening test for chromosomal aneuploidies and sex determination (47). However, transplant recipients may have circulating not only maternal and fetal but also donor-derived cffDNA, potentially complicating test accuracy. Therefore, in several cases, the results of NIPT were not coherent with the ultrasound image in terms of sex assessment. Thus, limiting NIPT to screening for autosomes only is suggested (48, 49). For the same reasons, assessing fetal blood type rhesus status is not recommended (50).

## 8 Immunosuppression strategies in gestation

While pregnancy affects females' immune systems and can enhance graft tolerance, all transplant recipients must continue their immunosuppressive regimens. Good graft functionality is critical for the mother to keep and maintain the pregnancy intact and the fetus healthy.

**Figure 2:**
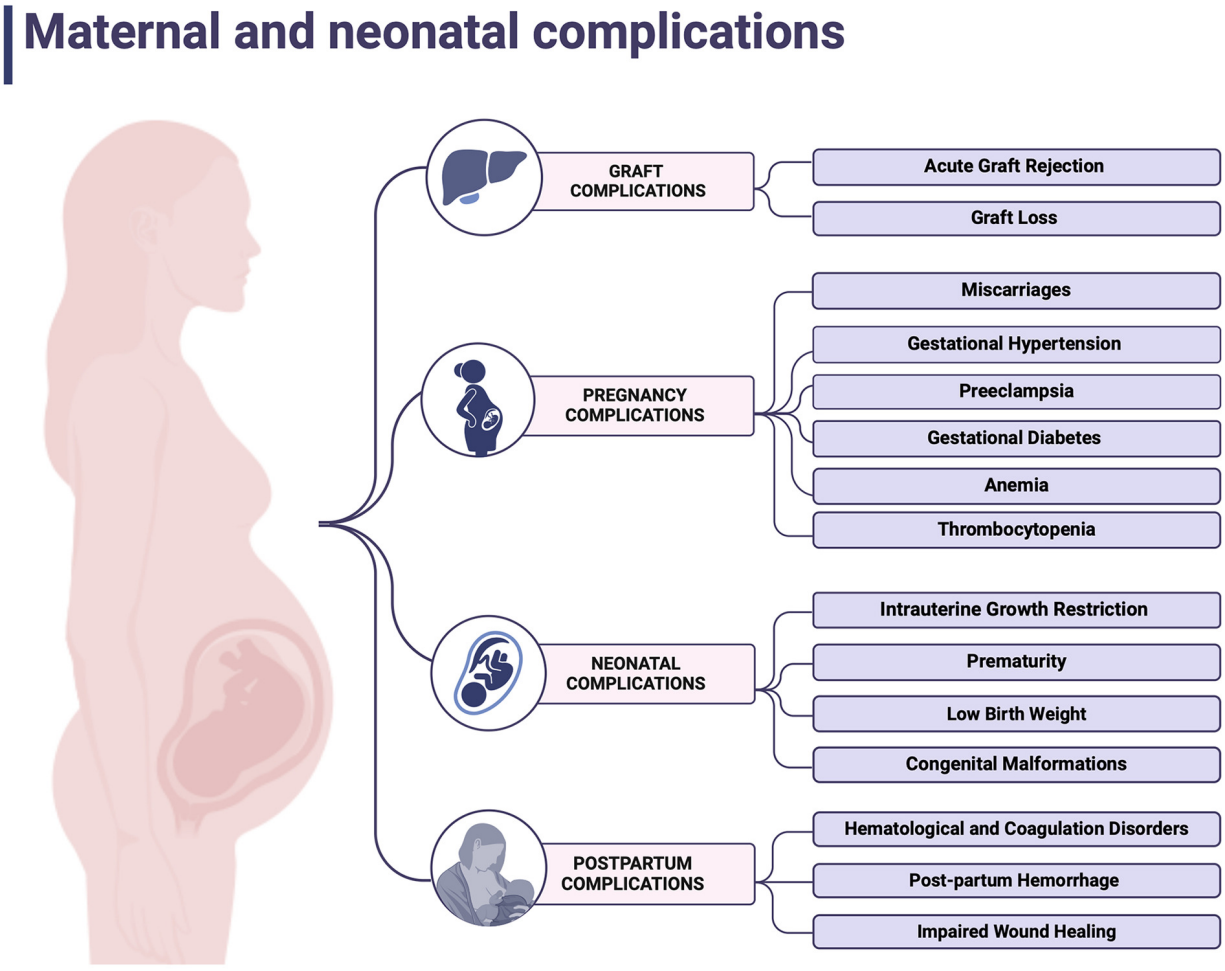
Safety profiles of immunosuppressive agents. Created with BioRender.com (53).

The American College of Gastroenterology recommends the need for temporal withdrawal of mycophenolate in OLT recipients as data on mTOR inhibitors outcomes are inconclusive (51). The best pregnancy outcomes are achieved when therapeutic schemes include calcineurin inhibitors (CNI) +/− corticosteroids +/− azathioprine, as confirmed by NTPR data (52). Safety profiles of immunosuppressive agents are summarized in Figure 2 (54).

### 8.1 Calcineurin inhibitors (CNI)

Calcineurin inhibitors (CNIs), including cyclosporine A and tacrolimus, are widely used in preventing graft rejection and autoimmune disease treatment. Both of these immunosuppressive drugs act by inhibiting calcineurin, leading to impairment of interleukin 2 release, which eventually results in the inhibition of T cell proliferation and cytotoxicity (55). Pregnancy alters drug metabolism by impacting hepatic cytochrome P450 enzymes, renal filtration and total body fluid volume. Thus, tacrolimus is metabolized by cytochrome P450 enzymes—CYP3A4 and CYP3A5 and binds to plasma proteins and erythrocytes. Hence, its active form is the unbound one, and its pharmacokinetics may be altered at gestation, adjusting the optimal dose requires more attention and precise monitoring. It should be based not only on plasma concentrations but also on red blood cells and albumin count (10, 56).

Recommendations provided by the AST assume tacrolimus serum levels of 3–10 ng/ml after 1 month from transplantation (57). According to SFMF guidelines, tacrolimus and cyclosporine concentrations during pregnancy should be screened monthly, starting at week 32, followed by either weekly or biweekly time-points and concluding once post-partum (11). FDA formerly classified CNIs as category C in pregnancy, meaning they should be used when the benefits outweigh the risks (58). In retrospective studies, tacrolimus use was associated with increased rates of preterm delivery (57%–59%) by cesarean section (34%–47%). No significant impact on the live birth rate was observed. Tacrolimus crosses the placenta, with a concentration in umbilical vein blood reaching 71 ± 18% of maternal blood serum concentration (59). Fetal malformations were observed in 5.6% of deliveries, but no specific pattern was discovered. Transient hypoxia, hyperkaliemia and renal dysfunctions were observed in neonates (58, 60). Cyclosporine is associated with similar outcomes to tacrolimus pregnancy outcomes. However, cyclosporine use was linked to a higher risk of developing gestational hypertension disorders than tacrolimus (61).

### 8.2 Azathioprine (AZA)

Azathioprine, metabolized by the liver to its active agent 6-mercaptopurine, inhibits lymphocytes proliferation by blocking the purine metabolism. AZA is currently being replaced by newer drugs but remains a therapeutic option. In terms of adverts events, it affects the gastrointestinal tract and may lead to vomiting and diarrhea episodes. Moreover, it can cause the suppression of bone marrow, resulting in leukopenia, anemia and thrombocytopenia (62). Azathioprine use in pregnancy has been associated with an increased risk of low birth weight and preterm delivery. Although no significant link to congenital defects has been established, the study on 476 patients treated with AZA revealed an increased malformation rate in this group compared to the general population (OR: 1.41, 95% CI: 0.98–2.04) (63). Therefore, decisions regarding its use at gestation should be taken individually by the attending physician, considering both grafts and fetus safety.

### 8.3 Mycophenolic acid (MPA) derivatives

Mycophenolate acid derivatives, among them mycophenolate mofetil (MMF), are strongly forbidden during pregnancy and are recommended to be discontinued six weeks before conception. Females of reproductive age taking MMFs are advised to use effective contraception. Mycophenolic acid acts by blocking purine synthesis in T and B lymphocytes. Outcomes of animal studies raised concerns about its intrauterine toxicity and teratogenic potential (64).

According to a study by Sifonitis et al., out of 26 pregnancies exposed to MMF, 15 resulted in live birth and in 4 babies birth defects were demonstrated, including microtia, cleft lip and palate, diaphragmic hernia, hypoplastic nails and heart defects (65). Further studies investigated if there is any pattern of congenital malformations after MMS *in utero* exposure. Recurring abnormalities included cleft lip and palate, microtia, external auditory canal atresia, micrognathia, and hypertelorism, followed by internal organs and brain defects (66).

Due to limited evidence specific to OLT, data from the National Transplantation Pregnancy Registry on female kidney transplant recipients indicate that switching from MPA regimens before conception significantly reduces the incidence of miscarriages and neonatal birth defects. Discontinuation of this medication and immediate substitution is strongly advised in unintended pregnancy situations (67).

### 8.4 Corticosteroids

Corticosteroids, the steroid hormone group that impacts immune response by modulating gene expression, are a widely used group, not only among transplant recipients. They act by suppressing the production of proinflammatory cytokines and activation of immune cells (67). Interestingly, their potential to promote the maturation of fetal lungs was found to have therapeutic use in antenatal care. As corticosteroids are a big group of pharmaceutic agents, their metabolism and further effect depend on the route, dose, and specific substance used. While generally well-tolerated, long-term use may result in glucose metabolism disturbances, hypertension, osteoporosis, mental health conditions, and impaired wound healing. Because of that, pregnant females require closer attention to the above-mentioned symptoms (68). Patients undergo liver cytochrome P450 enzymes transformations and renal elimination. As discussed before, pregnancy affects fluid volume and binding protein concentrations. Thus, the pharmacokinetics of corticosteroids may be altered. OLT recipients are typically treated with oral prednisone, metabolized by placental 11β-hydroxysteroid dehydrogenase type 2, which significantly limits its placental transfer and impact on the fetus (53, 69). Current literature has not established a significant correlation between corticosteroid uptake and birth defects. The only malformation after the use of corticosteroids in pregnant patients that increased rate was noticed were orofacial clefts (70).

### 8.5 Mammalian target of rapamycin (mTOR) inhibitors

Little has been studied in the literature regarding the use of mTOR inhibitors (sirolimus and everolimus) in pregnancy. These macrolide antibiotics block the cytokine-driven proliferation of lymphocytes T. Animal studies have shown that mTOR inhibitors may alter fetus development, its growth and even lead to intrauterine death (71). Blood serum levels of mTOR inhibitors are not associated with its therapeutic potential. Data available in the literature is case based only, and patients were exposed to other therapeutics at that time. AASD recommendations, based on NTPR data, discourage its use due to a high miscarriage rate, up to 31% and five reported cases of congenital malformation, mainly facial abnormalities and microtia (10, 65). Although mTOR inhibitors are primarily used in renal rather than liver transplant recipients, the lack of specific data in the latter group underscores the need for further investigation to guide clinical practice.

### 8.6 Belatacept

Betalacept is the fusion protein that acts as a T cell co-stimulation blocker. It was designed to replace CNIs due to their nephrotoxicity in renal transplant recipients, but included here for completeness, though rarely used after OLT. Belatacept is being used in conjunction with MPA agents and prednisone (53, 72). It is administered by monthly intravenous infusions. The most frequent adverse effects include gastrointestinal tract disturbances, anemia, elevated blood pressure, fever, and urinary tract infection (73). Animal studies have not demonstrated the risk of birth defects. However, higher pup mortality among rodents was noticed. Unfortunately, there is only minimal evidence of its use in gestation. Data from 16 cases documented by the NTPR indicated 13 live births and 3 miscarriages, with no reported congenital anomalies (74). However, the study group was insufficient and long-term outcomes are unknown. Therefore, belatacept is not recommended during pregnancy, and a transfer to acceptable agents is recommended before conceiving (53).

## 9 Intrapartum management of OLT recipients

Pregnancies following an OLT carry an elevated risk of complications, making delivery in a unit with an adequately prepared neonatal intensive care unit essential. While transplantation itself is not an indication for elective cesarean section (CS) or induction of labor (IoL), complications such as hypertension, preeclampsia, and gestational diabetes often require earlier delivery. The rate of elective CS among all transplant recipients is high and ranges from 7.3% to 67.4% (75). The mean rate of CS in population-based studies, reaches 57.9% which is 2.9-fold more than observed in non-transplant recipients (45). According to numerous experts, liver graft localization in the right upper abdomen does not allow for any harm during labor. Intraoperative injury during emergency CS is very unusual, and its prevalence was calculated for 0.27% in kidney recipients, yet among OLT recipients, it occurs even less frequently (76). A cohort retrospective study based on TPRI data showed a high number of non-medically indicated surgical deliveries come from physicians and patients' concerns about graft injury, vaginal delivery, infections, and neonatal and mother well-being. Clinical practice shows that some centers refer every transplant recipient for scheduled CS as part of their internal protocol.

A study by Yin et al. indicates that vaginal delivery does not negatively impact transplanted liver function or maternal outcomes. Additionally, it may lower the risk of neonatal respiratory distress (75). Decisions on delivery time and mode should be taken individually considering the general patient's situation and especially the potential risks.

In terms of medication, patients on long-term corticosteroids may be considered for additional stress doses during labor, as suggested by SFMF guidelines, but the evidence is weak and inconsistent, with no clear proof of their necessity (11, 77). Surgical delivery is advised to be under antibiotic prophylaxis, followed by thromboembolism risk assessment. Antibiotic prophylaxis is recommended for surgical deliveries. It should be followed by a thromboembolism risk assessment and preventive medical assessment if needed. Unless contraindicated, standard anesthesia and analgesia protocols can be implemented, including epidural and regional anesthesia for pain management (11).

## 10 Weighing the odds: maternal complications in OLT recipients

**Figure 3:**
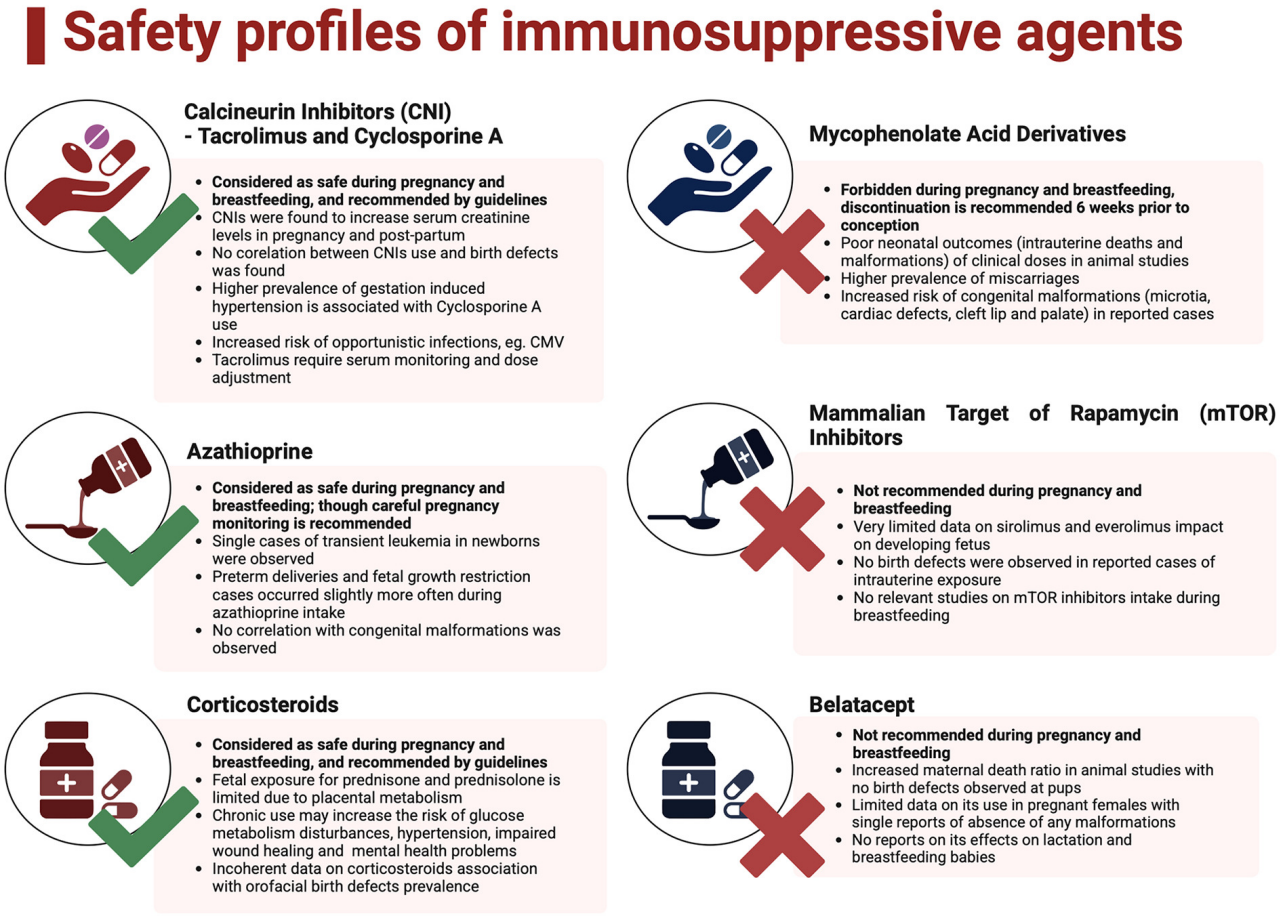
Maternal and neonatal complications. Created with BioRender.com.

A population-based study comparing OLT recipients' outcomes to the general US population, based on 7,288,712 deliveries, among them 2.1/100,000 were at women who underwent OLT, revealed a significantly higher risk of developing hypertensive disorders, gestational diabetes, anemia, and thrombocytopenia. Genitourinary tract infections, chorioamnionitis and impaired wound healing, occurred notably more often (45). In addition, analysis by Thornton et al. found that abruptions occur with a significantly higher prevalence than in American females (78). Deshpande et al. reveals also a problem of renal insufficiency that may occur pre-pregnancy and worsen through pregnancy (79, 80). Moreover, the incidence of labor induction and cesarean sections was higher than in the overall population. In terms of post-partum complications, increased amounts of hemorrhages, coagulopathies, and blood transfusion had remarkably more prominent prevalence (45). Both maternal and neonatal complications were summarized in Figure 3 (81).

### 10.1 Pregnancy complications

#### 10.1.1 Gestational hypertension and preeclampsia

The prevalence of hypertensive disorders of pregnancy differs regionally. However, the global median is 116.4 per 100,000 women of reproductive age (82). World Health Organization estimated that 14% of maternal deaths worldwide were associated with hypertension (83). Although its etiology is unclear and related to multiple pathomechanisms, risk factors requiring increased attention include pre-existing hypertension, diabetes mellitus, obesity, maternal age greater than 35 and primary kidney diseases (84). According to the International Society for the Study of Hypertension in Pregnancy, gestational hypertension, formerly known as pregnancy-induced hypertension, is defined as newly diagnosed systolic blood pressure ≥140 mmHg and/or diastolic blood pressure (DBP) ≥90 mm Hg after 20 weeks gestation, without proteinuria and preeclampsia symptoms. Preeclampsia is diagnosed when gestation hypertension is followed by proteinuria or signs of organ impairment (neurological/hematological/pulmonary/liver impairment/acute kidney injury) or uteroplacental dysfunction after 20 weeks gestation (85). Due to underlying disease, medication, and different organ malfunctions, OLT recipients are more vulnerable to developing hypertension during pregnancy. A review by Deshpande et al., based on 450 pregnancies in OLT recipients, proved that the preeclampsia rate pooled 21.9% and was higher than in the general population in the USA and Europe (45). Similarly, hypertension rates reached an incidence of 27.2% among graft recipients in comparison to 3.8% in the general population (80). It was confirmed by a population-based study by Ghazali et al., which revealed hypertensive disorders of pregnancy in 36.2% of recipients (45) and King's College experience with 19% of hypertension and 14% of preeclampsia cases among their post OLT patients (86). Gestational hypertension should be treated with antihypertensive medication allowed after OLT and in pregnancy as indicated by SFMF and NICE guidelines (11, 87). Prevalence of preeclampsia differs depending on immunosuppressive regimens, with the highest incidence in the cyclosporine-treated group reaching up to 68%–73% (10). If an OLT increases the risk for preeclampsia, its prevention with aspirin in all pregnant recipients requires consideration. Low-dose aspirin (initiated at 11–14 weeks and continued until 36 weeks) is widely recommended to mitigate preeclampsia risk by improving placental function (88). It is recommended by the Society for Maternal-Fetal Medicine and suggested by Rahim et al. guidance (11, 35).

#### 10.1.2 Gestational diabetes

According to NTPR data, gestational diabetes affects approximately 8% of pregnant OLT recipients, representing a 1.9-fold increased risk compared to the general populations, observed by Ghazali et al. (45). It must be outlined that graft recipients are predisposed to develop new-onset diabetes mellitus following transplantation, with prevalence ranging from 9% to 63%. Risk factors involve family history, hepatitis C virus infection, body mass index and immunosuppressive therapy (89). Medication, particularly CNIs and corticosteroids, significantly contributes to insulin resistance. Early glucose screening and vigilant management are critical to minimizing adverse outcomes (45).

### 10.2. Post-partum complications

#### 10.2.1 Organ rejection and graft loss

A cross-sectional analysis of the American population demonstrated that OLT rejection during the delivery time was observed in 4.1% of pregnant women (78). Data from the National Transplantation Pregnancy Registry (NTPR) reported a rejection rate of 5%, with OLT loss within 2 years post-delivery reaching 3% (10, 90). However, not all those losses and rejections were confirmed within liver biopsy. Cases of graft loss were very uncommon due to the possibility of pharmacological treatment with pulses of steroids and adjustment of immunosuppressive protocol. Significantly lower rejection rates were found in women who conceived more than 1-year post-transplantation, which was evaluated in King's College multi-center study on 117 pregnancies. Among them, 17 were diagnosed with acute cellular rejection during pregnancy or early post-partum. None of them lost the graft (10, 86). Whereas meta-analysis by Deshpande et al. reports acute liver rejection rate during pregnancy ranging from 5% to 17%, graft loss within 2 years of delivery occurred in 10.5% of patients (80).

#### 10.2.2 Hematological and coagulation disorders

Population based studies have demonstrated significantly higher prevalence of decreased red blood cells—and thrombocyte counts in females who underwent OLT (45). Hematological disturbances may be related to transplantation itself and caused by medication-induced myelosuppression, renal impairment, viral infections, and iron deficiency. Pregnancy and delivery may also affect blood components count in preeclampsia, post-partum hemorrhages followed by disseminated intravascular coagulation (45).

#### 10.2.3 Hemorrhage incidence

Post-partum hemorrhage in the Ghazali study occurred with a significant 3.2-fold incidence in OLT recipients, which was coherent with Coffin's discoveries. Authors claim that it may be associated with higher rates of cesarean sections, coagulation disturbances and decreased blood component count (45, 91).

## 11 Newborns of mothers who underwent OLT: health outcomes

Despite an elevated risk of neonatal complications, longitudinal studies have shown that transplant recipients can successfully deliver healthy neonates born at term. However, complications such as miscarriages, prematurity, low birth weight, and intrauterine growth restriction (IUGR) remain prevalent (45, 80, 86).

### 11.1 Miscarriage

A meta-analysis by Valentin et al., which included 38 studies encompassing 1131 pregnancies in 838 women post OLT, demonstrated a mean live delivery ratio of 80.4%. The miscarriage pooled rate was 16.7%, according to Valentin et al. (92). These findings are consistent with a review by Deshpande et al., based on 450 pregnancies, which reported a live birth rate of 76.9% and a miscarriage rate of 15.6% (80). Interestingly, live birth and miscarriage rates within the whole American population during the same period, as reported by the US National Vital Statistics Reports, were 66.7% and 17.1%, respectively (80). These data suggest that, with appropriate medical management, women who have undergone OLT can achieve live births rates comparable to the general population.

### 11.2 Intrauterine growth restriction (IUGR)

Fetal growth restriction (FGR), previously referred to as IUGR, is diagnosed via ultrasound when the estimated fetal weight is below the 10th percentile for gestational age. Etiological factors may be fetal, placental, or maternal, with a calculated prevalence ranging from 3% to 9% in developed countries (93). A comparative study of OLT recipients and the general population revealed a 4.1-fold higher likelihood of FGR in transplant recipients, likely attributed to a greater prevalence of hypertension, preeclampsia, and medications that may impair fetal growth (45).

### 11.3 Preterm delivery

Preterm delivery is defined as birth before 37 + 0 weeks of gestation. In a population-based study by Ghazali et al., preterm delivery was reported in 30% of pregnancies among OLT recipients, representing a 4.7-fold increase compared to the general population (45). It was confirmed by the Valentin et al. meta-analysis, with a mean rate of 32.1% preterm births, with pooled gestation age of 36 + 5 weeks (92). The literature, however, does not specify how many of these deliveries are the result of iatrogenic interventions by cesarean section. Thus, maternal complications, including preeclampsia, renal insufficiency or graft rejection, may play a pivotal role in a decision to deliver the pregnancy preterm.

### 11.4 Low birth weight

Low birth weight (LBW), defined as a weight below 2,500 g at birth, is an important indicator of a neonate's health and possible risks. According to Valentin et al., the mean birth weight of neonates born to OLT recipients was 2,691 g (92). Similarly, Deshpande et al. reported a pooled mean birth weight of 2,866 g, compared to the mean birth weight of 3,298 g observed in the general American population (80). King's College findings were also consistent (pooled weight −2,745 g), however, it was outlined that 29% of infants were born with low or very low (<1,500 g) weight at delivery, what was associated with risk of admission to a special care baby unit. LBW was a result of higher rates of preterm deliveries and incidence of hypertensive disorders resulting in placental disturbances (86).

### 11.5 Birth defects

Available reviews and meta-analyses are incoherent in terms of congenital malformations. The only study that found a significantly higher prevalence of birth defects among OLT recipients was population-based research prepared by Ghazali et al. (45). According to this data, 2% of recipients' infants would have a birth defect whereas among non-recipients, the prevalence of malformations reached 0.4%. However, due to a lack of documentation, scientists could not correlate it to any immunosuppressive regimen. A study based on NTPR data by Coscia et al. revealed comparable to general population incidence of congenital malformations among all organ recipients, including liver, unless they were exposed to MMF. In this situation, the occurrence of defects reached 23%. Reported anomalies do not follow any specific pattern and relate to different systems (pyloric stenosis, hypospadias, pulmonary venous stenosis anomaly) (52, 80). Other comprehensive reviews by Valentin et al. and Deshpande et al. have not revealed a significant association between congenital malformations in offspring and prior OLT (80, 92).

## 12 Post-partum care and long-term health of OLT recipients

The early post-partum period typically focuses on efficient pain management, breastfeeding and assessment of wound healing and graft functions. Pain control typically involves acetaminophen or non-steroidal anti-inflammatory drugs. However, medication interactions and dosing regimens require verification and adjustment according to liver and kidney functions. Routine antibiotic prophylaxis is not recommended unless there are clinical indications. An obstetric follow-up appointment should be scheduled 6 weeks post-partum, assessing maternal well-being, mental health and wound healing. At this appointment, the safety of breastfeeding and future contraception methods ought to be discussed. Additional follow-up provided by the transplant team is needed to evaluate liver functions and modify medication (94). Transplant recipients are primarily seriously ill patients; therefore, their long-term outcomes may depend more on underlying disease than pregnancy itself. One of the most extensive available studies, presenting Kings College's experience, revealed that out of 79 OLT recipients, who were pregnant, after pooled 52 months after the delivery, three women died. However, their deaths were not associated with pregnancy. Eight women required retransplantation after 18–120 months since pregnancy. However, none of these were due to the impact of pregnancy (86).

## 13 Post-OLT breastfeeding challenges

The WHO highlights the benefits of exclusive breastfeeding for the first six months after delivery (95). However, maintaining the transplanted organ in good condition requires the use of immunosuppressive medications for the rest of the one's life. While breastfeeding was historically discouraged for transplant recipients, the overall attitude of counseling physicians has changed lately, and it is now increasingly accepted. Mothers should be fully informed about the benefits and potential risks of lactation while on immunosuppressive therapy and make their own decisions (96). Counseling physicians should rely on the latest evidence published in peer-reviewed databases to provide accurate guidance.

One source of best practices is “LactMed, the Drugs and Lactation Database' available online, provided by the National Library of Medicine, as it contains valuable insights regarding the levels of medications in maternal milk and its impact on newborns based on all papers published worldwide (97). Drug excretion and accumulation in human milk depend on its chemical and physical properties, half-life and patient's metabolism. Some of those medications would turn into non-active metabolites before exertion. Recent years of research have expanded our understanding of the impact of immunosuppressive agents on breast milk. The literature points towards several modalities that are considered safe for neonates, including calcineurin inhibitors (tacrolimus and cyclosporine), azathioprine and corticosteroids, (particularly prednisone). However, mycophenolic acid derivatives, mTOR inhibitors, and belatacept are not deemed safe, and mothers are discouraged from breastfeeding while taking them (11).

### 13.1 Calcineurin inhibitors—tacrolimus and cyclosporine

Research on cyclosporine levels detected in breastfed infants' serum is not coherent. While cyclosporine has been detected in breast milk and infants' blood, the estimated exposure is approximately 2% of the maternal intake. Respectively, no side effects were observed in infants. Therefore, cyclosporine is claimed to be safe in lactation (98, 99). According to both transplant and rheumatology societies, as well as NTPR, tacrolimus penetration to milk is marginal, and mothers might be encouraged to maintain lactation. Though there is no data on complications in exposed kids, hence it was recommended in the literature that more long-term studies should be conducted (11, 96, 100, 101).

### 13.2 Azathioprine

Azathioprine (AZA) is considered safe by guidelines provided by transplant and rheumatology societies (96, 102). Maternal doses of up to 200 mg daily resulted in very low or undetectable medication concentrations in the milk and infant's serum. Therefore, it is suggested to avoid breastfeeding within 4 h after the last dose. There is no evidence of AZA in exposed newborns' blood serum. No symptoms of immunosuppression or any other significant short-term adverse effects were noticed in the infants (103). There is, however, one study evaluating long-term outcomes of 15 children exposed to AZA, with pooled age on follow-up at 3.3 years. In those children compared to the unexposed control group, no development disturbances and increased risk of infections and hospitalizations were observed (104).

### 13.3 Corticosteroids

The corticosteroid, most frequently used in OLT recipients, is prednisone in doses of 5–10 mg daily. Its concentration in breast milk is negligibly low, less than 0.1% of mothers' intake, which was revealed by conducted research (105, 106). No evidence has been found in terms of adverse effects on exposed neonates, which was also confirmed by NTPR's analysis of 169 infants (105, 107). Women are advised to breastfeed at least 4 h after taking the medication to minimize infants' exposure. However, there are single case reports suggesting the impact of high doses of corticosteroids on transient inhibition of lactation. It was observed that triamcinolone given intraarticularly inhibited milk production and ejection. Lactation was finally restored with domperidone, a dopamine antagonist medication (108).

### 13.4 Mycophenolic acid derivatives

Since mycophenolic acid agents are not used during pregnancy, breastfeeding is strongly discouraged in mothers taking this substance. There is insufficient data regarding its concentration in breast milk. According to the NTPR, seven women continued mycophenolate during lactation without significant adverse effects on their children. However, 2 out of 7 babies resulting from pregnancies where mycophenolic acid was used by the mother were born with congenital malformations (11, 65, 96, 109).

### 13.5 mTOR inhibitors and belatacept

Limited data are available on the effects of mTOR inhibitors and belatacept during pregnancy and lactation. These agents are typically used in combination with other immunosuppressive medications. Animal studies demonstrated that both drugs are passed to breast milk. However, due to the lack of reliable evidence in the literature and potential risks to the newborn, breastfeeding is not recommended in this clinical setting (11, 96).

## 14 Summary of recommendations: take-home message

This review outlines the risks and challenges associated with family planning following OLT, including preconception guidance, pregnancy oversight, and management of complications. Key studies in this field were summarized in Table 2, @ (7, 52, 90, 12, 19, 10, 11, 25, 80, 96, 45, 35, 92, 61, 50, 53, 51, 110, 86, 58, 78, 75, 91)while Table 3 compares the pregnancy care in a healthy and OLT recipient populations. Accordingly, following evidence-based strategies are recommended to achieve the best maternal and neonatal outcomes:

• **Preconception counseling**: All reproductive-age transplant recipients should be counseled during the perioperative phase to discuss contraception, optimal conception timing (preferably 12–24 months post-transplant), pregnancy risks, and necessary health adjustments.
• **Immunosuppressive therapy management:** Regimens require modifications prior to conception, as some of used medications should be discontinued due to their teratogenic risks (mycophenolate acid-based agents). These should be switched to safer alternatives like tacrolimus or azathioprine.
• **Multidisciplinary team:** Care should be provided by a team of hepatologists, obstetricians, transplant surgeons, and neonatologists, from preconception through the post-partum period.
• **Careful pregnancy monitoring:** Frequent evaluations of liver function, immunosuppressive drug concentrations, and maternal-fetal health need to be performed with regular visits to screen for common complications like gestational hypertension, preeclampsia, or fetal growth restriction.
• **Post-partum care and breastfeeding support**: Graft function must be closely monitored after delivery to ensure no signs of rejection are detected. Patients should be guided through breastfeeding, with safe medications tacrolimus, azathioprine, prednisone being promoted and high-risk agents (mycophenolic acid derivatives, mTOR inhibitors) being avoided.

**Table 2:** Key studies on pregnancy and family planning post-transplantation.

Year	Authors	Summary	Conclusions	Ref.
Registry reports				
2007	Sibanda et al.	Provides initial data on maternal and fetal outcomes.	Pregnancy is feasible post-transplantation with a majority resulting in live births.	(7)
2010	Coscia et al.	Summarizes outcomes of pregnancies in transplant recipients, from the NTPR data with a focus on maternal and neonatal outcomes.	Points the role of registries collecting data that form clinical practice and future guidelines.	(52)
2017	Moritz et al.	The annual report from TPRI on pregnancy outcomes in transplant recipients provides new data and trends.	Underlines the importance of registry data for improving clinical practices in managing pregnancy after OLT.	(90)
Guidelines				
2005	McKay et al.	Summarizes the findings from the AST Consensus Conference on reproductive issues in transplantation.	Provides criteria for safe pregnancy planning and management. Highlights the need for individualized care.	(12)
2016	Nguyen et al.	Recommendations for the contraceptive methods use in women with various medical conditions, including OLT.	Guidelines for safe contraception in transplant recipients, considering their health, medication and graft risks.	(19)
2021	Sarkar et al.	Evidence-based recommendations on reproductive health for patients with liver disease, including transplantation.	Recommendations emphasize the importance of preconception counselling and the need for a multidisciplinary approach.	(10)
2023	Irani et al.	Detailed guidelines for prepregnancy evaluation and pregnancy management in solid organ transplant recipients, including liver-specific risks.	Emphasizes the importance of careful prepregnancy evaluation, stable allograft function, pregnancy monitoring, and immunosuppressive therapy management.	(11)
Reviews				
2010	Paulen et al.	A systematic review on contraceptive use in solid organ transplant recipients, including the safety and efficacy of each method.	Emphasize the importance of contraceptive options for transplant recipients to prevent unplanned pregnancies.	(25)
2012	Deshpande et al.	A systematic review and meta-analysis of pregnancy outcomes in OLT recipients.	Pregnancy is feasible in OLT recipients but requires careful monitoring due to elevated complications risks.	(80)
2014	Constantinescu et al.	Evaluation of the safety of breastfeeding in transplant recipients during immunosuppressive therapy.	Breastfeeding with certain immunosuppressants is safe and possible; however, individualized patient counselling is required.	(96)
2017	Ghazali et al.	Population-based study analyzing pregnancy outcomes in OLT recipients, focusing on complications compared to general population data.	Identifies higher risks of complications like hypertension and preterm birth requiring specialized care.	(45)
2020	Rahim et al.	Review on pregnancy management, including preconception and delivery considerations.	Emphasizes preconception counselling and role of multidisciplinary team care for successful pregnancy outcomes.	(35)
2021	Valentin et al.	Systematic review and meta-analysis on pregnancy outcomes after OLT, assessing live birth rates and complications.	Noted high live birth rates, but also a elevated prevalence of risks, such as preterm delivery.	(92)
2022	Akiyama et al.	Meta-analysis on pregnancy and neonatal outcomes in women during CNI therapy.	Provides evidence on the safety of calcineurin inhibitors during pregnancy.	(61)
2022	Kallapur et al.	Review on care strategies for pregnancy in solid organ transplant recipients.	Emphasizes the role of a multidisciplinary care team and close monitoring.	(50)
2024	Saad et al.	Reviews the safety and management of immunosuppressive agents before and during pregnancy in transplant recipients.	Outlines that some immunosuppressants are safe during pregnancy and that adherence to prescribed regimens is crucial to prevent graft rejection.	(53)
2024	Kothari et al.	Update of clinical guidance on managing pregnancy-related liver diseases, including OLT.	Current recommendations for pregnancy management in patients post OLT.	(51)
2025	Katz-Greenberg et al.	Updated, evidence-based guidelines reflecting the latest clinical practices across all solid organ transplants.	The review concludes that while pregnancy is feasible in solid organ transplant recipients, it requires careful planning and management.	(110)
Single-center studies				
2015	Westbrook et al.	Report on single-center outcomes of pregnancy post-OLT.	Provides data on the safety of pregnancy post-transplantation.	(86)
2019	Kamarajah et al.	A single-center study examining maternal and neonatal outcomes in OLT recipients.	Provides evidence of successful pregnancies with specialized care and monitoring.	(58)
2021	Thornton et al.	Investigates obstetric outcomes in OLT recipients, focusing on complication rates.	Notes increased risks of hypertension, emphasizing the need for specialized obstetric care.	(78)
2021	Yin et al.	Analysis of delivery modes and outcomes in kidney and OLT recipients over five decades.	Vaginal delivery was found safe and potentially beneficial, reducing neonatal respiratory distress.	(75)
Nationwide case-control analysis				
2010	Coffin et al.	Nationwide case-control study of pregnancy outcomes in OLT recipients, including maternal and neonatal risks, compared to general population data.	OLT recipients and their infants face increased risks of obstetric complication, but most pregnancy outcomes are favorable.	(91)

**Table 3:** Pregnancy care: healthy population vs. OLT Recipients.

Aspect of care	General population	OLT recipients
Prenatal screening and genetic counseling	Standard screening (e.g., NIPT, 20-week scan).	Standard screening (e.g., NIPT, 20-week anomaly scan).
	Genetic counseling based on age, family history, or screening results; no transplant-related concerns.	Genetic counseling if liver disease is hereditary (e.g., Wilson’s disease, hemochromatosis).
		Additional focus on teratogenic drug risks.
Immunosuppression management	Not applicable.	Regular monitoring and potential adjustment of immunosuppressive therapy to balance graft tolerance and fetal safety. Requires consultation with a transplant specialist.
Graft function monitoring	Not applicable	Frequent liver function tests (e.g., ALT, AST, bilirubin) and possibly imaging studies to ensure graft health throughout pregnancy.
Infection risk	Standard prenatal infection screening.	Enhanced infection screening, including tests for opportunistic infections.
Specific risks	Gestational hypertension: 3%–5%.	Gestational hypertension: ∼27%
	Preeclampsia: 5%–8%.	Preeclampsia: ∼22%
	No graft or immunosuppression-related risks.	Graft rejection: ∼4% during pregnancy.
		Increased infection risk due to immunosuppression.
Fetal monitoring	Standard ultrasounds and fetal heart rate monitoring	More frequent ultrasounds and possibly specialized tests to monitor fetal growth and well-being. Lower threshold for intervention if fetal distress is detected.
Delivery considerations	Cesarean rate ∼20%–30%, typically for obstetric indications (e.g., breech, labor dystocia).	Cesarean rate ∼58% due to maternal/fetal complications (e.g., hypertension, graft instability).
	Vaginal delivery standard unless complications arise.	Vaginal delivery feasible if no complications, though often avoided due to caution.
Postpartum care	Standard postpartum checkups focusing on recovery and breastfeeding	Additional monitoring for graft rejection, infection, and overall health. Close follow-up with obstetrician and transplant team. Breastfeeding may require special consideration due to medications.

## 15 Conclusions and future challenges for pregnancy after OLT

The desire to have children is a fundamental biological and emotional need engraved by evolution, and no one should be deprived of this opportunity. Advances in medical science, including organ transplantation, have transformed once life-threatening diseases into manageable conditions. OLT serves as a prime example, enabling many young women to regain hormonal balance, fertility, and a normal quality of life. These women can now live, work, love, and, for many, pursue their desire to conceive and start a family. International perinatal and transplant societies provide comprehensive guidelines for pre-pregnancy counseling and pregnancy management in post-transplant patients. Recent research has also expanded to explore the impact of transplantation on fertility, the use of assisted reproductive technologies, and the development of birth control strategies. Moreover, there are preliminary studies on the long-term effects of in-utero and breastfeeding-related exposure to immunosuppressive therapies on child development.

Nevertheless, this field remains underexplored. Unanswered questions include: What are the optimal birth control methods, particularly hormonal options, and how can their safety and efficacy be better evaluated? Why is there a higher prevalence of pregnancy complications in transplant recipients, and what preventive strategies can be developed to mitigate these risks? Finally, how do immunosuppressive therapies affect infants in the long-term, and what adjustments are needed to optimize breastfeeding recommendations?

Available literature and observational studies consistently demonstrate that women post-OLT can achieve successful pregnancies and delivering healthy full-term infants. However, all the evidence is primarily based on retrospective and observational studies, as ethical constraints prevent the conduct of interventional trials in pregnant women. Despite these limitations, existing data have allowed us to identify the most common maternal and neonatal complications, as well as obstetric risks associated with delivery in this unique patient group. In sum, these studies offer hope that with careful management, successful pregnancies following OLT may be achievable. Future research is needed to further optimize outcomes and guide clinical practice.
